# Experiences of quality cluster meetings in general practice – Findings from a national survey two years after initiation of quality clusters in Denmark

**DOI:** 10.1186/s12875-025-02759-4

**Published:** 2025-03-03

**Authors:** Maria Bundgaard, Line Bjørnskov Pedersen, Jens Søndergaard, Marius Brostrøm Kousgaard, Sonja Wehberg, Dorte Ejg Jarbøl

**Affiliations:** 1 Research Unit of General Practice, Department of Public Health, University of Southern Denmark, Campusvej 55, Odense M, 5230 Denmark; 2 Danish Centre for Health Economics - DaCHE, Department of Public Health, University of Southern Denmark, Campusvej 55, Odense M, 5230 Denmark; 3 The Research Unit for General Practice, Department of Public Health, University of Copenhagen, Øster Farimagsgade, København K, 1014 Denmark

**Keywords:** General practice, Primary health care, Quality improvement, Quality circles, Quality of health care, Surveys and questionnaires, Cross-sectional study, Denmark, Self administrated questionnaire

## Abstract

**Background:**

A new national model for quality improvement in general practice based on the concept of quality clusters was introduced in Denmark in 2018. A quality cluster is a local group of general practitioners (GPs) meeting regularly to engage in quality improvement on self-selected topics.

**Aim:**

To explore (1) GPs’ experiences of cluster meetings, and (2) associations between meeting experiences and self-reported benefits of participation.

**Design:**

A national cross-sectional survey study in general practice. In 2020, a questionnaire regarding quality clusters was sent to all Danish GPs (*n* = 3432). GPs self-reported benefits from cluster participation comprised: overall benefit, changes in clinical organization and workflow, changes in drug prescriptions, improved knowledge of guidelines, and improved patient care.

**Results:**

1219 GPs (36%) participated. Results showed that cluster meetings were partly or fully perceived to be well organized (89%) and focused on relevant topics (89%), and that meetings took place in a friendly atmosphere (90%) where experiences were shared (93%). Two-thirds of the GPs found that the data was useful (67%), that their cluster showed a high level of commitment (66%), and that agreement was easily reached (61%). Meetings which were perceived as productive, with useful data, and with a high level of commitment were associated with statistically significantly higher odds for reporting benefits across all self-reported benefits investigated.

**Conclusion:**

Overall, cluster meetings were perceived positively by the GPs and associated with benefits when experienced as productive, with useful data, and a high level of commitment.

**Supplementary Information:**

The online version contains supplementary material available at 10.1186/s12875-025-02759-4.

## Key message

In general, the GPs evaluated the cluster meetings positively

30% of the GPs reported high or very high overall benefit from cluster participation

Two out of three of the GPs found that data was useful

Productive meetings, useful data, and a high level of commitment was mostly associated with benefit

It is important to continue with supporting the clusters in obtaining relevant data

**Supplementary Information:**

The online version contains supplementary material available at 10.1186/s12875-025-02759-4.

## Introduction

In 2018, a new model for quality improvement in general practice, referred to as quality clusters, was introduced in Danish general practice by the Organization of General Practitioners in Denmark and the Danish Regions via the collective agreement [1]. The Danish quality clusters consist of local groups of general practitioners (GPs) committed to meet regularly and work with quality improvement on self-selected topics in a data-driven way [1]. The cluster meetings constitute the focal point of the improvement concept.

Recent studies of Scottish and Welsh equivalents to the Danish clusters have pointed to some start-up difficulties including lack of engagement among the participating GPs and challenges with facilitation, leadership, and use of data [2–5]. Knowledge from learning and improvement initiatives in small professional groups in a general practice setting, recommends that meetings in the groups should be perceived as safe, with open discussions, and a supportive environment where the atmosphere is neither too cozy nor too threatening [6–9]. A recent study of the Danish clusters found that approximately 70% of the participating GPs reported moderate to very high overall benefit from cluster participation and that clusters with relatively active (having plenum discussions and groupwork) and frequent (3–6 yearly) meetings were positively associated with reported benefits [10]. Nearly all Danish GPs (approximately 98%) registered in a cluster and after the initial phase with formally setting-up the clusters, the focus has now turned to making the meetings attractive as a place for learning and development [11, 12].

To further investigate the impact and mechanisms of quality clusters we wish to explore (1) the GPs’ experiences of cluster meetings, and (2) the associations between selected GP meeting experiences and their self-reported benefits, comprising: (a) overall benefit, (b) changes in clinical organization and workflow, (c) changes in drug prescriptions, (d) improved knowledge of guidelines, and (e) improved overall patient care in the clinic.

As the previous paper [10] addressed the organisation of the clusters and how this is associated with the doctors’ perceived benefits of cluster participation, this study addresses what happens at the meetings and whether the meeting aspects are perceived as beneficial. This will strengthen our understanding of the concept and its potential impact on quality improvement and consequently reveal areas in need of focus, support, or adjustments.

## Materials and methods

### Institutional setting

Danish GPs are self-employed and remunerated through taxes in a mixed capitation (approximately 30%) and fee-for service system (approximately 70%) [13, 14]. There were 3315 self-employed GPs working under the collective agreement in 2021, and salaried GPs (employed doctors or locums) constituted around 6% of the work force in Danish general practice [15, 16]. Every second year, the GPs’ collective agreement with the public funder is renegotiated. The agreement details terms and requirements concerning various aspects of the work in general practice such as types of services provided, remuneration fees, and continuous medical education, and quality improvement strategies, now including the quality clusters [13, 14].

The Danish clusters are constituted by GPs from the same geographical area (with a mean size of 34 GPs per cluster, ranging from 10 to 68 GPs) that meet on a regular basis (typically 3–4 times a year) to engage in quality improvement work [10]. The GP practices registered in clusters between April 2018 and October 2019. Management of the clusters is decentralized, and the GPs have autonomy to decide which quality improvement topics to engage with and how. Remuneration is only available for GPs with administrative tasks in the cluster, such as cluster coordinators or board members. According to the collective agreement, the clusters should work to improve quality in a data-driven way, i.e. by using relevant data [1]. This can be data on services provided, medical treatment, or referrals, comparing diagnostics, prescribing rates and referrals between the participating GP clinics. In each cluster, the overall responsibility for the management of the cluster rests with the cluster coordinator [1, 11, 17]. The cluster coordinator is elected by and from the group of GPs in the cluster and is often supported by some administrative cluster members (the coordinator and the administrative members are referred to as cluster leads). The cluster meetings vary in content with most using plenum discussions, group work, professional presentations, clinical guidelines, and clinical data to facilitate peer discussions [10]. Sometimes, representatives from the surrounding healthcare sector, e.g. the local hospital or municipality, are invited to the meetings [10]. To support the clusters, various cluster packages with topic specific material exist [10, 11].

### Study design

Two years after the introduction of the concept, we conducted a cross-sectional survey study to explore different aspects of the Danish quality clusters. The questionnaire was part of the second wave of a GP work-life survey sent yearly to all Danish GPs. Administrative data on GPs, practices, and clusters were obtained from other sources detailed below.

### Study population

The study population comprised all GPs in Denmark with an identifiable practice provider number i.e. being a practice owner working under the collective agreement. Employed GPs or GPs working as locums were excluded from the study as they are not inherent cluster members. Due to risk of ambiguous information on practice characteristics and cluster organization, GPs with more than one provider number were excluded.

### Questionnaire development

To achieve content and face validity, the questionnaire was developed and tested in four steps [18]: (1) a literature search exploring concepts that were somewhat similar to the Danish clusters and particularly inspired by literature of the Scottish clusters [2, 4, 8, 9, 19–22], (2) qualitative interviews with cluster leads and ordinary GP cluster members, (3) a phase of conceptualization and operationalization, and (4) a pilot test with 3 GPs (two cluster members and one cluster coordinator) where time consumption, content validity, acceptability, and feasibility were tested. The development of the questionnaire is described in detail in Bundgaard et al. 2022 [10].

The final questionnaire exploring quality clusters consisted of two parts. The first part covered questions about the organization of the clusters and were given to leading cluster members only. The second part explored the GPs’ experiences and benefits of engaging in cluster meetings as well as each GP’s role in the cluster (ordinary or leading member) and degree of participation in meetings. The second part was given to all GPs including leading members and informs the present paper.

### Survey distribution

The survey was distributed in June 2020 through postal mail together with an information letter containing a hyperlink and a personal code to the online questionnaire. An electronic regional newsletter informing about the up-coming survey was distributed to all five regions in Denmark. Two postal reminder letters were sent out in July and August, respectively. The cluster leads were additionally reminded by e-mail and telephone. The survey was closed in December 2020.

### Administrative data

Three publicly available administrative data sources were used to obtain information on GPs and their practices: (1) The practice address, region, and provider number were obtained from The Danish Health Data Network (MedCom) [23, 24], (2) practice type (singlehanded or partnership practice) and GP name were obtained from The Danish eHealth Portal (Sundhed.dk) [25], and (3) GP age from The Danish Health Professional registry (Autorisationsregisteret) governed by the Danish Ministry of Health. Cluster information was received from the cluster supporting organization, Quality in General Practice (KiAP), in September 2020, and included practice provider number of registered practices in the cluster, date of membership, and information on the cluster coordinator. The background information was linked with survey responses through OPEN/REDCAP [26–28] using the unique practice provider number.

### Variables and statistical approach

*Explanatory variables.* Experience of cluster meetings were measured on a five-point Likert scale (fully disagree, partly disagree, neither nor, partly agree, to fully agree) in the following eleven items: (1) topics are relevant, (2) meetings are productive, (3) atmosphere is friendly, (4) discussions are fruitful, (5) data are useful (for the quality improvement in the GP clinics), (6) meetings are well organized, (7) meeting duration is suitable, (8) practice data are discussed openly, (9) experiences are shared, (10) agreement is easily reached, and 11) commitment is high. Definitions of the variables are provided in Table A1 Codebook, placed in the supplementary material. All statistical analyses are performed in Stata 17.

*Outcomes.* We measured the self-reported benefit from participating in clusters with five items: (1) overall benefit, (2) changes in the clinical organization and workflow, (3) changes in drug prescriptions (drug type or dose), (4) improved knowledge of guidelines, and (5) improved overall patient care in the clinic. Overall benefit was measured on a five-point Likert scale (1 = very little, 2 = little, 3 = moderate, 4 = high, 5 = very high). The remaining items were measured using a four-point Likert scale (1 = none, 2 = little, 3 = to some degree, 4 = to a high degree) with the possibility of answering “not relevant” (responses in “the not relevant” category were excluded from the regression analysis).

We estimated the associations between GPs’ experiences from participating in the cluster meetings and their perceived benefit in five separate multivariable ordered logit regression models at the individual GP level using cluster robust standard errors at the quality cluster level. Each of the items of self-reported benefits were included as dependent variables and the GPs’ meeting experiences were included as binary explanatory variables dichotomized into (1) fully agree and partly agree, versus (2) fully disagree, partly disagree and neither/nor. The models were adjusted for GP characteristics (age and gender), practice type (single-handed or partnership practice), area (region), and selected cluster organizational characteristics including meeting frame (frequency, duration, rules for attendance) and content (use of plenum discussions, groupwork, cluster packages, and guidelines). The selected cluster organizational variables were identified as significant in a preceding study exploring the associations between cluster organization and benefit [10]. In addition, we adjusted for upstart (time between practice membership in a cluster and survey answer), size (number of practices in a cluster), GP role (ordinary member or cluster lead), and degree of participation (participated in few, half, most, or all cluster meetings). An overview of all data used in the study with exact questions posed, response scales, coding of variables, and data sources are provided in a codebook in Table A1 in the supplementary material.

Missing values for the adjusting variables (displayed in Table A2) were allocated to the majority group. There were no missing values for the explanatory variables.

We further provide descriptive statistics for the dependent variables (self-reported benefits) and the explanatory variables (meeting experiences). These are also stratified by membership status and placed in the supplementary, Table A5 and A6. In the supplementary material we also provide tables with results from the regression analyses stratified on membership status. The analysis plan was designed prior to the analyses.

## Results

### Study population

**Fig. 1:**
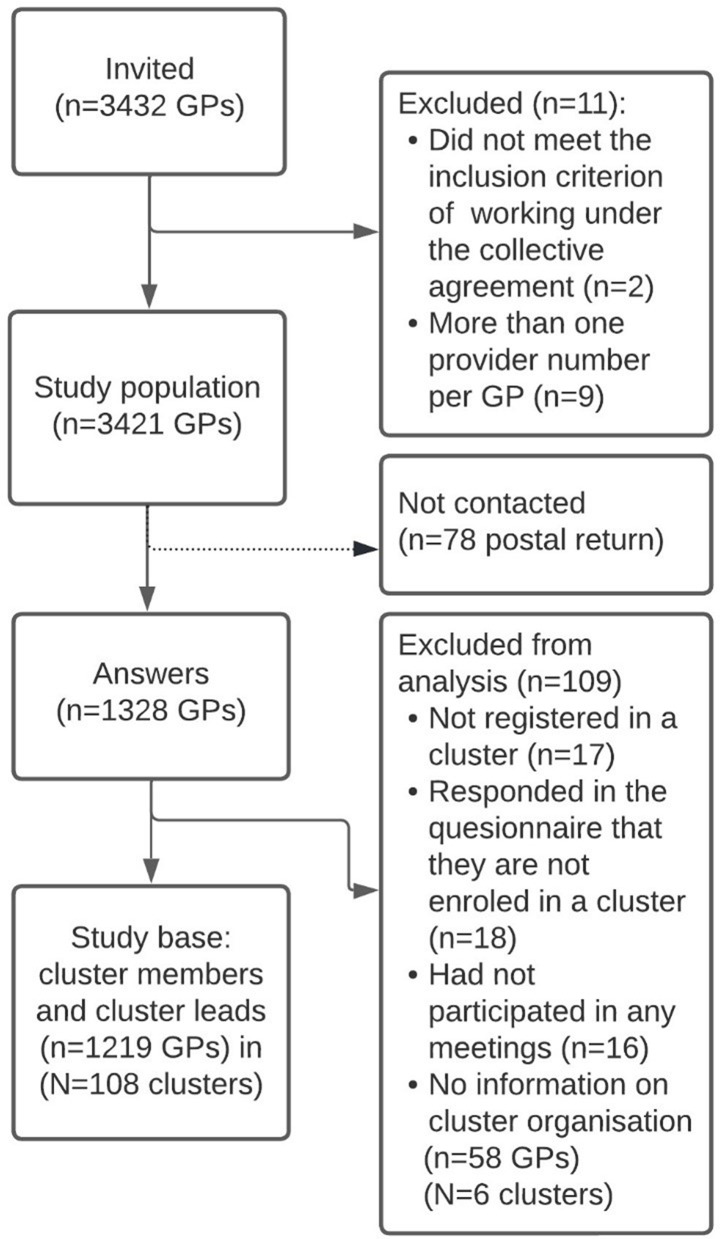
Flowchart of the study population

The study base consisted of 1219 GPs corresponding to 36% of the invited GP population. Leading cluster members accounted for 220 out of the 1219 answers, representing 108 out of 114 clusters in total. The study population is illustrated in Fig. 1. Characteristics of the study base, the non-respondents, and the total Danish GP populations are shown in Table 1. The responders resemble the non-responders well with respect to gender (*p* = 0.622) and practice type (*p* = 0.778). However, age (*p* < 0.001) and regional distribution (*p* < 0.001) showed statistically significant differences with higher representation of younger GPs from the Southern and Central regions of Denmark.

**Table 1:** Characteristics of the general practitioner population, survey respondents, and non-respondents, 2020

Characteristics	GP population in DK	Respondents	Non-respondents	*P*-value
Total, n (%)	3421 (100.0)	1219 (100.0)	2202 (100.0)	
**GP gender**, n (%)				0.622
Male	1453 (42.5)	524 (43.0)	929 (42.2)	
Female	1952 (57.1)	688 (56.4)	1264 (57.4)	
Missing	16 (0.5)	7 (0.6)	9 (0.4)	
**GP age in years**, **n (%)**				< 0.001
<46	1039 (30.4)	367 (30.1)	672 (30.5)	
46–55	1142 (33.4)	453 (37.2)	689 (31.3)	
>55	1172 (34.3)	373 (30.6)	799 (36.3)	
Missing	68 (2.0)	26 (2.1)	42 (1.9)	
**Practice type**, **n (%)**				0.778
Singlehanded	809 (23.6)	285 (23.4)	524 (23.8)	
Partnership	2611 (76.3)	934 (76.6)	1677 (76.2)	
Missing	1 (0.0)	0 (0.0)	1 (0.0)	
**Region**, **n (%)**				< 0.001
Capital Region	1079 (31.5)	338 (27.7)	741 (33.7)	
Zealand	446 (13.0)	166 (13.6)	280 (12.7)	
Southern Denmark	790 (23.1)	309 (25.3)	481 (21.8)	
Central Denmark	804 (23.5)	316 (25.9)	488 (22.2)	
Northern Denmark	282 (8.2)	84 (6.9)	198 (9.0)	
Missing	20 (0.6)	6 (0.5)	14 (0.6)	

### Experience of cluster meetings

Table 2 shows that overall, the reaction to working in clusters was positive. Cluster meetings were partly or fully perceived to be well organized (89%), and focused on relevant topics (89%), and that meetings take place in a friendly atmosphere (90%) where experiences are shared (93%). Two-thirds of the GPs partly or fully agreed that the data was useful (67%), and that there was a high level of commitment (66%), and that agreement was easily reached (61%).

**Table 2:** Perceived meeting experience in clusters answers from 1219 GPs, 2020

	Fully disagree *n* (%)	Partly disagree *n* (%)	Neither nor *n* (%)	Partly agree *n* (%)	Fully agree *n* (%)
Topics are relevant	6 (0.5)	51 (4.2)	74 (6.1)	384 (31.5)	704 (57.8)
Meetings are productive	30 (2.5)	106 (8.7)	160 (13.1)	486 (39.9)	437 (35.8)
Atmosphere is friendly	5 (0.4)	33 (2.7)	86 (7.1)	354 (29.0)	741 (60.8)
Discussions are fruitful	14 (1.1)	87 (7.1)	156 (12.8)	459 (37.7)	503 (41.3)
Data are useful	40 (3.3)	110 (9.0)	253 (20.8)	510 (41.8)	306 (25.1)
Meetings are well organized	5 (0.4)	33 (2.7)	100 (8.2)	429 (35.2)	652 (53.5)
Meeting duration is suitable	15 (1.2)	49 (4.0)	67 (5.5)	414 (34.0)	674 (55.3)
Practice data are discussed openly	20 (1.6)	56 (4.6)	146 (12.0)	409 (33.6)	588 (48.2)
Experiences are shared	8 (0.7)	18 (1.5)	59 (4.8)	383 (31.4)	751 (61.6)
Agreement is easily reached	25 (2.1)	86 (7.1)	344 (28.2)	532 (43.6)	232 (19.0)
Commitment is great	24 (2.0)	103 (8.4)	292 (24.0)	470 (38.6)	330 (27.1)

### Self-reported benefits

Table 3 shows the GPs’ self-reported benefits from cluster participation. Overall benefit from cluster participation was perceived to be moderate by 40%, high by 25%, and very high by 5% of the responding GPs.

**Table 3:** Self-reported benefits answers from 1219 GPs, 2020

	Very little *n* (%)	Little *n* (%)	Moderate *n* (%)	High *n* (%)	Very high *n* (%)
Overall benefit	123 (10.1)	241 (19.8)	488 (40.0)	305 (25.0)	62 (5.1)
	None n (%)	Little n (%)	To some degree n (%)	To a high degree n (%)	Not relevant n (%)
*Changes in*:					
Clinical organization and workflow	366 (30.0)	398 (32.6)	365 (29.9)	61 (5.0)	29 (2.4)
Drug prescriptions	251 (20.6)	401 (32.9)	408 (33.5)	139 (11.4)	20 (1.6)
*Improvements*:					
Increased knowledge of guidelines	379 (31.1)	394 (32.3)	339 (27.8)	63 (5.2)	44 (3.6)
Overall patientcare in the clinic	259 (21.2)	559 (45.9)	328 (26.9)	51 (4.2)	22 (1.8)

### Associations between GP reported benefits and experience of cluster meetings

**Fig. 2:**
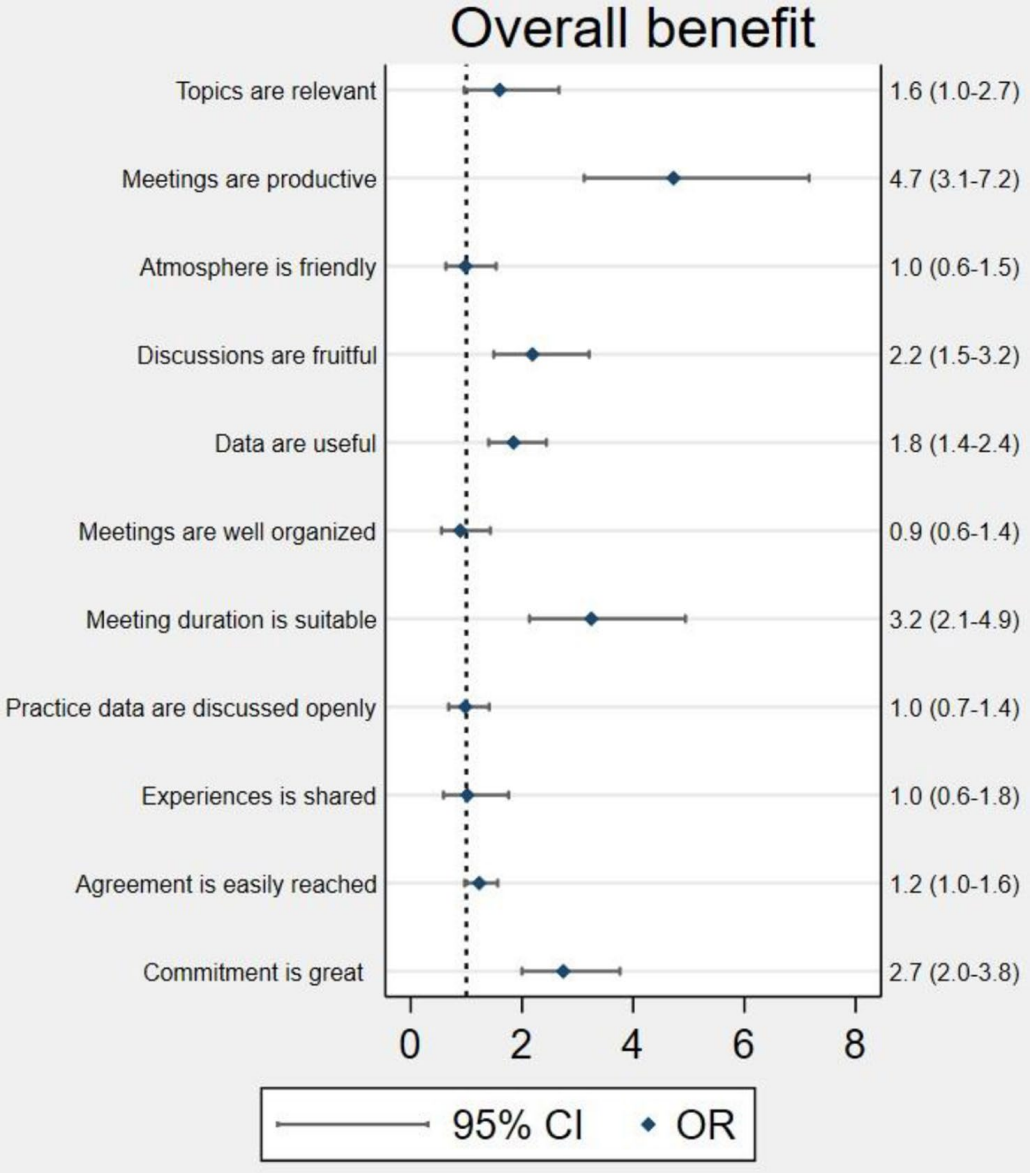
Associations beween cluster meeting experience and self-reported overall benefit answers from 1219 GPs, 2020. Figure notes: Ordered logistic multivariable regression model with cluster robust standard errors at the quality cluster level. Adjusted for GP age and gender, practice type, area, cluster organizational characteristics (frame: frequency, duration, and rules for attendance; content: use of plenum discussions, groupwork, cluster packages, and guidelines), upstart date, size, GP role, and degree of participation in meetings. Missing values were allocated to the majority group for categorical variables: female, The Capital Region and the mean for continuous variables: mean GP age was 51 years

**Fig. 3:**
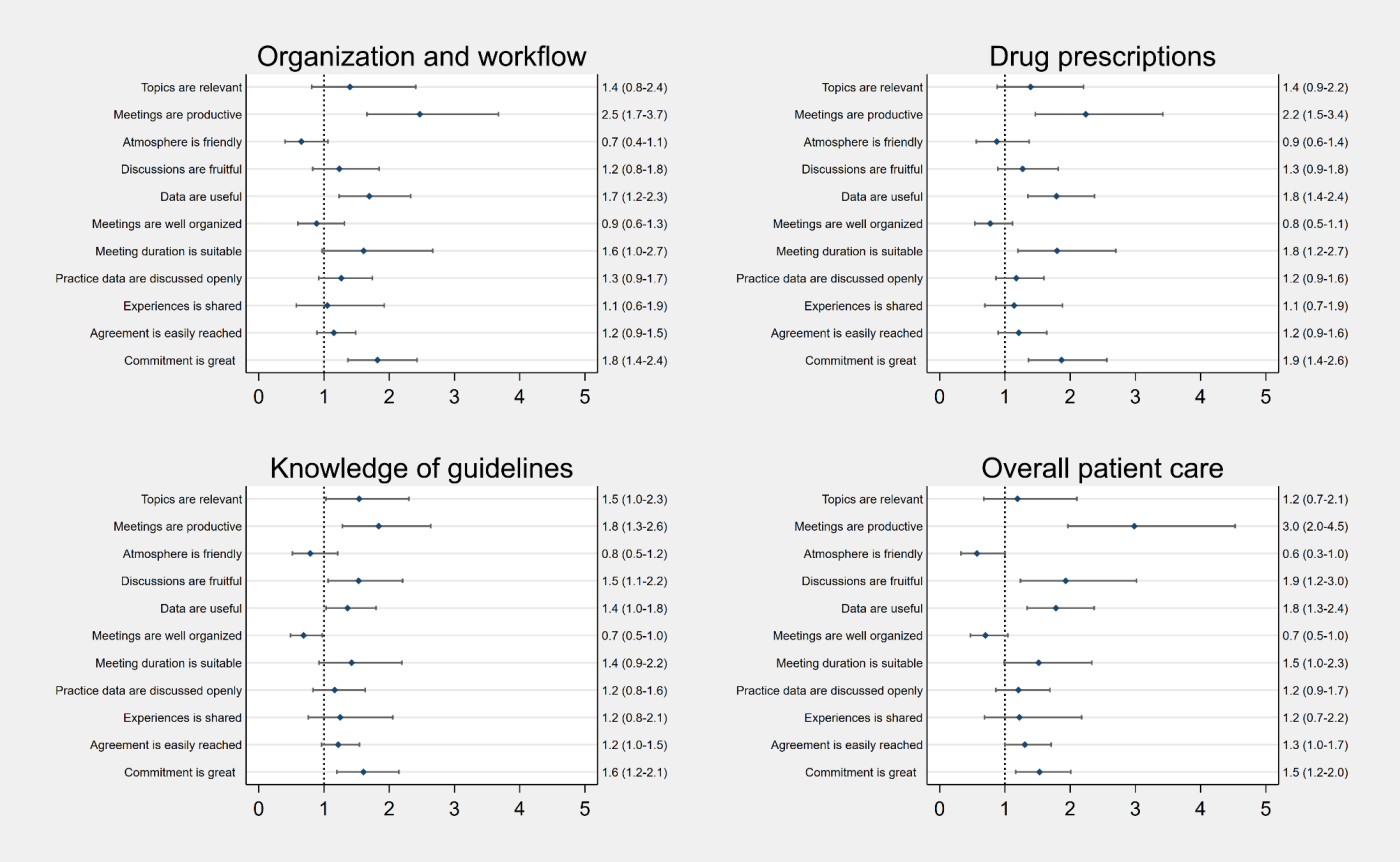
Associations between cluster meeting experience and self-reported clinical changes and improvements, 2020. Figure notes: Ordered logistic multivariable regression model with cluster robust standard errors at the quality cluster level. Adjusted for GP age and gender, practice type, area, cluster organizational characteristics (frame: frequency, duration, and rules for attendance; content: use of plenum discussions, groupwork, cluster packages, and guidelines), upstart date, size, GP role, and degree of participation in meetings. Missing values were allocated to the majority group for categorical variables: female, The Capital Region and the mean for continuous variables: mean GP age was 51 years. Number of respondents are reported in table A4 and A5 in the supplementary

All associations between GPs’ self-reported benefits from cluster participation and meeting experiences are shown in Figs. 2 and 3 and in the corresponding Tables A2-A4.

Figure 2 shows the associations between GPs’ perceived overall benefit and meeting experience.

Statistically significantly higher odds for overall benefit of participating in a cluster were reported from GPs that experienced the meetings as productive (OR 4.7; CI 3.1–7.2), and with fruitful discussions (OR 2.2; CI 1.5–3.2), having useful data (OR 1.8; CI 1.4–2.4), with a suitable meeting duration (OR 3.2; CI 2.1–4.9), and a high level of commitment (OR 2.7; CI 2.0-3.8).

Figure 3 shows statistically significantly positive associations between self-reported changes in the clinical organization and workflow for GPs who find the meetings productive, with useful data, and a high level of commitment (OR 2.5; CI 1.7–3.7, OR 1.7; CI 1.2–2.3 and OR 1.8; CI 1.4–2.4, respectively). The same variables are also significantly associated with changes in drug prescriptions. In addition, suitable meeting duration (OR 1.8; CI 1.2–2.7) was also significantly associated with changes in drug prescription. Increased knowledge of guidelines was positively associated with relevant topics (OR 1.5; CI 1.0-2.3), productive meetings (OR 1.8; CI 1.3–2.6), fruitful discussions (OR 1.5; CI 1.1–2.2), useful data (OR 1.4; CI 1.0-1.8), and a high level of commitment (OR 1.6 CI; 1.2–2.1). Improved overall patient care in the clinic was also positively associated with productive meetings, fruitful discussions, useful data, and a high level of commitment.

Notably there was a negative association between increased knowledge of guidelines and experiencing the cluster meetings as well organized. Although not statistically significant, the same tendency was seen for the other outcomes. There was also a tendency of negative associations between having a friendly atmosphere at the meetings and several of the outcome measures. The robustness checks estimating separate regression analysis for each of the two groups, i.e. ordinary cluster members and cluster leads separately, showed that the cluster leads tend to be more positive (higher ORs), but in all cases in the same direction as the ordinary cluster members not leading to any marked differences in the overall analysis (Supplementary A7-A9).

## Discussion

### Summary of findings

The vast majority of the responding GPs experienced the meetings as having relevant topics, friendly atmospheres, and being well-organized, with a suitable duration, and a sharing of experiences. Fewer, but still two out of three of the GPs, experienced that data at the meetings were useful, that commitment at the meetings was high, and that it was easy to reach agreement at the meetings. More than two thirds of the GPs reported overall benefits from cluster participation. Between 31 and 45% reported to have made changes in the clinical organization and with drug prescribing in the clinic and improvements in their knowledge of guidelines and in the overall patient care. The meeting characteristics most frequently positively associated with self-reported benefits were productive meetings, useful data, and high commitment. On the other hand, experiencing a friendly atmosphere, and well-organized meetings tended to be negatively associated with reported benefits.

### Strengths and limitations

While it is an advantage of the study that the total GP population in Denmark was invited, it is a limitation that only 36% could be included for analysis. Even though the respondents did not differ on gender and practice type there were differences between age and region, and we cannot rule out non-responder bias. If, for example, the responding GPs are more engaged and positive towards the clusters this could lead to an overestimation of positive experiences and benefits. Incorporating the questionnaire in a larger survey with other topics might have accommodated such potential response bias. Data was collected in 2020 two years after the initiation of the quality clusters, therefore representing the start-up period of the clusters. The survey was sent out shortly after the first wave of the COVID-19 pandemic, during which shutdowns led to a pause in cluster meeting activities for a period until virtual meetings became available. This may have affected the responses and introduced a risk of recall bias as it might have been some time since the respondents had participated in a meeting. Despite these limitations there are probably knowledge relevant to countries with similar health care systems when implementing concepts of quality improvement in primary care.

Since we were interested in the significance and size of odds ratios between specific features of meeting experiences and benefits, we chose a non-statistically driven approach when selecting the explanatory variables of interest. The selection was performed on basis of expert judgement, key points from the literature, the qualitative pre-study, and central elements from the cluster concept.

We decided to perform the analysis on all responding GPs, regardless of their role in the cluster. By keeping the responses from the 220 cluster leads in the analyses, measured experiences and benefits may be positively biased since the cluster leads have a special responsibility for the activities of the clusters. However, reasoning that the cluster leads are also GPs and therefore part of the target group for the cluster intervention, we decided to keep them in the main analysis. The answers to questions of meeting experience were predominantly positive (60–93% of GPs partly or fully agreed to the questions). This entails that some variables have a low degree of variation, which introduces a risk of not having enough power in the statistical models to identify significant associations. On the other hand, having a significance level at 0.05 entails that 5% of the significant results are expected to be due to pure randomness.

We intentionally refrained from defining the term ‘overall benefit’, as we aimed to allow the GPs to provide their subjective assessment of the overall benefit of the cluster work. Positive experience of cluster meetings and higher benefits are close to each other in terms of wording and understanding and therefore at risk of being correlated. This is especially true when being measured at the same time as in a questionnaire study. However, we wanted to explore what features were most associated with self-reported benefits and we did find variations in associations between the features suggesting that correlation was not equally pronounced, making this limitation less of a problem for our analyses. Moreover, including these questions enabled comparison on meeting experiences and experienced benefits with a Scottish survey study of GP clusters [4]. We did not find large variation in which features that were significantly associated with benefits across the five selected outcome measures. Even though the five different measures selected are not exhaustive, the results indicate that it is the same meeting features that are associated with positive benefits from working in the clusters across different types of benefits, e.g. changes in drug prescriptions or improved knowledge of guidelines.

We believe that the results are of both national and international interest for the future organisation of the quality clusters.

### Discussion of findings and comparison with existing literature

Research about quality circles, that consist of smaller groups of peers (approx. 6–12), has suggested that facilitators of such groups should strive to ensure a friendly and relaxed atmosphere to promote the sharing and discussion of material and topics [9].

In the study of GP clusters from Scotland, cluster leads reported that their cluster meetings were perceived as friendly and well organized, but not always productive [4]. Compared to the Scottish results we find that most of the Danish GPs experienced that the meetings had a friendly atmosphere, and that the meetings were productive and well-organized. Although we find positive associations between productive meetings and fruitful discussions and self-reported benefits, we find tendencies of negative associations between well-organized meetings and meetings with a friendly atmosphere and self-reported benefits. This suggests that cozy meetings are not sufficient for GPs to experience benefits, and that well-organized meetings do not necessarily ensure benefits, hypothetically because they do not always allow for fruitful discussions.

Use of data is a key element in both the Scottish and Danish cluster models. The evaluation of the Scottish clusters concluded that more support to the cluster leads in data access and data analysis was needed to gain full potential of the GP cluster concept in Scotland [4, 5, 29]. In Wales, an evaluation also concluded that the use of patient data was still a challenge that needed to be addressed, although the clusters had been introduced in 2014 [2, 3]. In our study we found an association between experiencing data as useful and reporting positive benefits of cluster participation. We also found that two out three GPs perceived data to be useful, while one out of eight did not find data useful. In Denmark the cluster coordinators are ultimately responsible for presenting relevant data at the cluster meetings. A Danish interview study found that collecting relevant data for cluster meetings and facilitating peer discussions about data, was perceived to be challenging by the cluster coordinators [30]. Hence, providing support for cluster leaders in their efforts to collect, present, and analyse relevant data should be an ongoing focus area, and the cluster coordinator (or another designated person at the meeting) should have a good understanding of the data being discussed, e.g. in relation to questions about data validity or about variation between clinics. A recent Danish interview study explored how GPs in the clusters understand data from their own clinics, and what makes them trust or question a data analysis. The study highlights the importance of shifting from a data driven approach to a data informed approach [31].

Generally, many of the GPs responded *partly agree* and not *fully agree* to the questions about meeting experience. There may be various explanations for this. From a qualitative interview study with the cluster coordinators, we learned that there were differences in the sources of data and how the clusters collect data, depending on the topic, which may affect the data quality from meeting to meeting. For example, it is often easier to obtain good data on medication prescribing than it is on cooperation with the municipality. Furthermore, at some meetings, external data consultants have provided data for the cluster, while at other meetings the members themselves were responsible for collecting the data from their own electronic medical system, which can cause challenges [30]. Hence, different approaches and circumstances depending on the selected topic may explain why GPs are not fully satisfied with the cluster meetings all together.

Approximately two thirds of the respondents reported no change or improvements due to work in the cluster. While further research is required to explain this finding, we know from research on audit and feedback interventions that the effects of such interventions on clinical behavior and outcomes vary significantly [32, 33]. Thus, bringing about change in professional behavior via feedback on current practice has been shown to be a complex process influenced by a host of interacting factors (such as the existing evidence on best practice; professional performance at base line; leadership; the time, attitudes, and skills of professionals etc.) [33, 34], and this also applies to the cluster concept. It may also be explained by the novelty of the clusters at the time of study or the complexity of transferring knowledge from one setting to another e.g. changing behaviour that in this case also involves staff, agreeing with practice colleagues that may not have attended the meeting and convincing patients who may have other preferences.

## Conclusion and implications

Overall, the GPs evaluated the cluster meetings positively. Meetings experienced as productive, with useful data, and a high level of commitment was the most frequent characteristics positively associated with the five self-reported benefits of cluster participation. Although most GPs perceived data to be useful, the results also suggest that it is important to continue to improve support for the clusters in obtaining, analysing and facilitating data in the clusters. Supporting clusters obtaining and handling data would therefore be a recommendation for other countries having or establishing clusters like the Danish as this issue is also supported by findings from other countries with somewhat similar improvement concepts [35]. Furthermore, it would be a natural next step to investigate what leads to productive meetings, useful data, or high commitment as these elements seem important for experiencing benefits from cluster participation based on our findings.

## Electronic supplementary material

Below is the link to the electronic supplementary material.

Supplementary Material 1

## Data Availability

The datasets generated and analyzed in the current study are not publicly available and cannot be shared due to the data protection regulations of the Danish Data Protection Agency. Access to data is strictly limited to the researchers who have obtained permission for data processing. This permission was given to the Research Unit of General Practice, Department of Public Health, University of Southern Denmark.
