# Supplementary material for: Experiences of quality cluster meetings in general practice – Findings from a national survey two years after initiation of quality clusters in Denmark
Source: BMC Prim Care. 2025 Mar 3;26:63. doi: 10.1186/s12875-025-02759-4 (PMC11874380; doi:10.1186/s12875-025-02759-4)
Supplement: Supplementary file 1 — Supplementary Material 1 [file 12875_2025_2759_MOESM1_ESM.docx]

# Supplementary

| **Table A1. Codebook** | | | | |
| --- | --- | --- | --- | --- |
| **Variable name** | **Category** | **Data type** | **Definition** | **Operationalization (values)** |
| **GP reported effects (outcome)** |  | | | |
| Overall benefit |  | Self-reported only ordinary cluster members (by survey answer) | How is your overall benefit from working in quality cluster? | Very little (1), little (2), moderate (3), high (4),  very high (5) |
| Changes |  | Self-reported only ordinary cluster members (by survey answer) | Did the improvement work with specific topics in the cluster lead to changes in your practice regarding: |  |
|  | Clinical organization and workflow | Self-reported only ordinary cluster members (by survey answer) | Organization of work (e.g. workflow, delegation) | None (1), little (2), to some degree (3), to a high degree (4), not relevant (excluded) |
|  | Drug prescriptions | Self-reported only ordinary cluster members (by survey  answer) | The clinical work in relation to the prescriptions (e.g. choice of drug, dose). | None (1), little (2), to some degree (3), to a high degree (4), not relevant (excluded) |
| Improvements | Knowledge of guidelines | Self-reported only ordinary cluster members (by survey answer) | Did the improvement work with specific topics in the cluster give you improved knowledge of recommendation material and guidelines (from the authorities or professional consensus groups) | None (1), little (2), to some degree (3), to a high degree (4), not relevant (excluded) |
|  | Overall patient care in the  clinic | Self-reported only ordinary cluster members (by survey  answer) | Has cluster work improved the overall patient care in your clinic? | None (1), little (2), to some degree (3), to a high degree (4), not relevant (excluded) |
| **Explanatory variables: Experience of cluster meetings** |  | Self-reported only ordinary cluster members (by survey answer) | To what extent do you agree or disagree with the following statements about the cluster meetings |  |
| Topics are relevant |  | Self-reported only ordinary cluster members (by survey answer) | Topics are relevant | Fully disagree, partly disagree, neither nor (0) partly agree, fully agree (1) |

| Meetings are productive |  | Self-reported only ordinary  cluster members (by survey answer) | Meetings are productive | Fully disagree, partly disagree, neither nor (0) partly agree, fully agree (1) |
| --- | --- | --- | --- | --- |
| Atmosphere is friendly |  | Self-reported only ordinary cluster members (by survey  answer) | Atmosphere is friendly | Fully disagree, partly disagree, neither nor (0) partly agree, fully agree (1) |
| Discussions are fruitful |  | Self-reported only ordinary cluster members (by survey answer) | Discussions are fruitful | Fully disagree, partly disagree, neither nor (0) partly agree, fully agree (1) |
| Data are useful |  | Self-reported only ordinary cluster members (by survey answer) | Data are useful | Fully disagree, partly disagree, neither nor (0) partly agree, fully agree (1) |
| Meetings are well organized |  | Self-reported only ordinary  cluster members (by survey answer) | Meetings are well organized | Fully disagree, partly disagree, neither nor (0) partly agree, fully agree (1) |
| Meeting duration is suitable |  | Self-reported only ordinary cluster members (by survey answer) | Meeting duration is suitable | Fully disagree, partly disagree, neither nor (0) partly agree, fully agree (1) |
|  |  |  | To what extent do you agree or disagree  do you agree with the following statements about your cluster? |  |
| Practice data are discussed openly |  | Self-reported only ordinary cluster members (by survey  answer) | In our cluster, we talk openly about the presented practice data | Fully disagree, partly disagree, neither nor (0) partly agree, fully agree (1) |
| Experiences are shared |  | Self-reported only ordinary cluster members (by survey answer) | In our cluster, we share our experiences from our own practice | Fully disagree, partly disagree, neither nor (0) partly agree, fully agree (1) |
| Agreement is easily reached |  | Self-reported only ordinary cluster members (by survey answer) | In our cluster, it is easy to reach an agreement | Fully disagree, partly disagree, neither nor (0) partly agree, fully agree (1) |
| Commitment is great |  | Self-reported only ordinary  cluster members (by survey answer) | In our cluster there is a great deal of commitment | Fully disagree, partly disagree, neither nor (0) partly agree, fully agree (1) |
| **GP, practice, and cluster characteristics (adjusting variables)** |  | | | |
| GP | Age | Register data | GP age | Continuous |
|  | Gender | Register data | Male or female | Male (0), female (1) |
| Participation in meetings |  | Self-reported only ordinary cluster members (by survey  answer) | How many of the cluster meetings have you attended since joining the cluster. |  |

|  | Few | Self-reported only ordinary  cluster members (by survey answer) | Few of the meetings | No (0), yes (1) |
| --- | --- | --- | --- | --- |
|  | Half | Self-reported only ordinary cluster members (by survey  answer) | About half of the meetings. | No (0), yes (1) |
|  | Most | Self-reported only ordinary cluster members (by survey answer) | Most of the meetings. | No (0), yes (1) |
|  | All | Self-reported only ordinary cluster members (by survey answer) | All meetings | No (0), yes (1) |
| Practice type | Singlehanded or partnership | Register data | Singlehanded practice with one GP listed, and Partnership practice with more than one GP listed. | Singlehanded (0), partnership (1) |
| Region | Capital region | Register data | Capital region of Denmark | No (0), yes (1) |
|  | Zealand | Register data | Region of Zealand | No (0), yes (1) |
|  | Southern Denmark | Register data | Region of Southern Denmark | No (0), yes (1) |
|  | Central  Denmark | Register data | Region of Central Denmark | No (0), yes (1) |
|  | Northern Denmark | Register data | Region of Northern Denmark | No (0), yes (1) |
| Cluster characteristics | Size | Register data | Number of GPs registered in the cluster | Continuous |
|  | Upstart | Register data | Months from registered cluster membership of the practice to survey  answer | Continuous |
| Meeting frame | Frequency > 2 meetings | Self-reported from cluster leads (by survey answer) | How many cluster meetings do you have during the year (under normal circumstances)? | Meetings per year 1-2 (0),  '3-4, 5-6, >6 (1) |
|  | Duration and type "Mixed meetings" | Self-reported from cluster leads (by survey answer) | What kind of meetings have you had in the cluster? | Only out of hours meetings 2-3 hours (0), mixed out of hours and/or half day, all day, residency (1) |
|  | Rules | Self-reported from cluster leads (by survey answer) | Are there agreed rules for attendance in the cluster? | No (0), yes (1) |
| Meeting content | Plenum discussions | Self-reported from cluster leads (by survey answer) | Have you in one or more of the cluster meetings used the following? | Plenary discussions were not used (0), Plenary discussions were used (1) |
|  | Groupwork | Self-reported from cluster leads (by survey answer) |  | Groupwork was not used (0), groupwork were used (1) |

|  | Cluster package | Self-reported from cluster leads (by survey answer) | Have you in one or more of the cluster meetings used the following? | Cluster package was not used (0),  Cluster package from KiAP and/or other, e.g. the Regional Quality Unit, was used (1) |
| --- | --- | --- | --- | --- |
|  | Guidelines | Self-reported from cluster leads (by survey answer) | Have you in one or more of the cluster meetings used the following? | Guidelines were not used (0), Guidelines were used (1) |

### Table A2. Associations between cluster meeting experience and self-reported overall benefit answers from 1219 GPs, 2020

| Perceived meeting experiences | Overall benefit | P-value |
| --- | --- | --- |
| Topics are relevant | 1.6 (1.0-2.7) | 0.0723 |
| Meetings are productive | 4.7 (3.1-7.2) | <0.001 |
| Atmosphere is friendly | 1.0 (0.6-1.5) | 0.9409 |
| Discussions are fruitful | 2.2 (1.5-3.2) | <0.001 |
| Data are useful | 1.8 (1.4-2.4) | <0.001 |
| Meetings are well organized | 0.9 (0.6-1.4) | 0.6293 |
| Meeting duration is suitable | 3.2 (2.1-4.9) | <0.001 |
| Practice data are discussed openly | 1.0 (0.7-1.4) | 0.9130 |
| Experiences are shared | 1.0 (0.6-1.8) | 0.9516 |
| Agreement is easily reached | 1.2 (1.0-1.6) | 0.0879 |
| Commitment is great | 2.7 (2.0-3.8) | <0.001 |

Table notes:

Ordered logistic multivariable regression model with cluster robust standard errors at the quality cluster level. Adjusted for GP age and

gender, practice type, area, cluster organizational characteristics (frame: frequency, duration, and rules for attendance; content: use of

plenum discussions, groupwork, cluster packages, and guidelines), upstart date, size, GP role, and degree of participation in meetings.

Missing values were allocated to the majority group for categorical variables: female, The Capital Region and the mean for continuous

variables: mean GP age was 51 years.

### Table A3. Associations between cluster meeting experience and clinical changes with organization and drug prescription, 2020

|  | Changes |  |  |  |
| --- | --- | --- | --- | --- |
| Perceived meeting experiences | Organization and workflow n=1190 | P-value | Drug prescriptions n=1199 | P-value |
| Topics are relevant | 1.4 (0.8-2.4) | 0.2273 | 1.4 (0.9-2.2) | 0.1561 |
| Meetings are productive | 2.5 (1.7-3.7) | <0.001 | 2.2 (1.5-3.4) | <0.001 |
| Atmosphere is friendly | 0.7 (0.4-1.1) | 0.0845 | 0.9 (0.6-1.4) | 0.5629 |
| Discussions are fruitful | 1.2 (0.8-1.8) | 0.3025 | 1.3 (0.9-1.8) | 0.1853 |
| Data are useful | 1.7 (1.2-2.3) | 0.0012 | 1.8 (1.4-2.4) | <0.001 |
| Meetings are well organized | 0.9 (0.6-1.3) | 0.5490 | 0.8 (0.5-1.1) | 0.1725 |
| Meeting duration is suitable | 1.6 (1.0-2.7) | 0.0662 | 1.8 (1.2-2.7) | 0.0045 |
| Practice data are discussed openly | 1.3 (0.9-1.7) | 0.1492 | 1.2 (0.9-1.6) | 0.3060 |
| Experiences are shared | 1.1 (0.6-1.9) | 0.8720 | 1.1 (0.7-1.9) | 0.6034 |
| Agreement is easily reached | 1.2 (0.9-1.5) | 0.2796 | 1.2 (0.9-1.6) | 0.2115 |
| Commitment is great | 1.8 (1.4-2.4) | <0.001 | 1.9 (1.4-2.6) | <0.001 |

Table notes:

Ordered logistic multivariable regression model with cluster robust standard errors at the quality cluster level. Adjusted for GP age and

gender, practice type, area, cluster organizational characteristics (frame: frequency, duration, and rules for attendance; content: use of

plenum discussions, groupwork, cluster packages, and guidelines), upstart date, size, GP role, and degree of participation in meetings.

Missing values were allocated to the majority group for categorical variables: female, The Capital Region and the mean for continuous

variables: mean GP age was 51 years.

### Table A4. Associations between cluster meeting experience and improvements in knowledge of guidelines and overall patient care

**in the clinic, 2020**

| Improvements | | | | |
| --- | --- | --- | --- | --- |
| Perceived meeting experiences | Knowledge of guidelines n=1175 | P-value | Overall patient care in the clinic n=1197 | P-value |
| Topics are relevant | 1.5 (1.0-2.3) | 0.0355 | 1.2 (0.7-2.1) | 0.5412 |
| Meetings are productive | 1.8 (1.3-2.6) | <0.001 | 3.0 (2.0-4.5) | <0.001 |
| Atmosphere is friendly | 0.8 (0.5-1.2) | 0.2787 | 0.6 (0.3-1.0) | 0.0508 |
| Discussions are fruitful | 1.5 (1.1-2.2) | 0.0222 | 1.9 (1.2-3.0) | 0.0038 |
| Data are useful | 1.4 (1.0-1.8) | 0.0296 | 1.8 (1.3-2.4) | <0.001 |
| Meetings are well organized | 0.7 (0.5-1.0) | 0.0338 | 0.7 (0.5-1.0) | 0.0812 |
| Meeting duration is suitable | 1.4 (0.9-2.2) | 0.1089 | 1.5 (1.0-2.3) | 0.0567 |
| Practice data are discussed openly | 1.2 (0.8-1.6) | 0.3783 | 1.2 (0.9-1.7) | 0.2746 |
| Experiences are shared | 1.2 (0.8-2.1) | 0.3817 | 1.2 (0.7-2.2) | 0.4941 |
| Agreement is easily reached | 1.2 (1.0-1.5) | 0.1015 | 1.3 (1.0-1.7) | 0.0509 |
| Commitment is great | 1.6 (1.2-2.1) | 0.0015 | 1.5 (1.2-2.0) | 0.0022 |

Table notes:

Ordered logistic multivariable regression model with cluster robust standard errors at the quality cluster level. Adjusted for GP age and

gender, practice type, area, cluster organizational characteristics (frame: frequency, duration, and rules for attendance; content: use of

plenum discussions, groupwork, cluster packages, and guidelines), upstart date, size, GP role, and degree of participation in meetings.

Missing values were allocated to the majority group for categorical variables: female, The Capital Region and the mean for continuous

variables: mean GP age was 51 years.

### Table A5. Perceived meeting experience in clusters; answers divided by cluster membership status, 2020

|  | Fully disagree n (%) | | | Partly disagree n (%) | | | Neither nor n (%) | | | Partly agree n (%) | | | Fully agree n (%) | | |
| --- | --- | --- | --- | --- | --- | --- | --- | --- | --- | --- | --- | --- | --- | --- | --- |
|  | All | OCM | CL | All | OCM | CL | All | OCM | CL | All | OCM | CL | All | OCM | CL |
| Topics are relevant | 6 (0.5) | 6 (0.6) | 0 | 51 (4.2) | 49 (4.9) | 2 (0.9) | 74 (6.1) | 72 (7.2) | 2 (0.9) | 384 (31.5) | 344 (34.4) | 40 (18.2) | 704 (57.8) | 528 (52.9) | 176 (80.0) |
| Meetings are productive | 30 (2.5) | 30 (3.0) | 0 | 106 (8.7) | 103 (10.3) | 3 (1.4) | 160 (13.1) | 140 (14.0) | 20 (9.1) | 486 (39.9) | 401 (40.1) | 85 (38.6) | 437 (35.8) | 325 (32.5) | 112 (50.9) |
| Atmosphere is friendly | 5 (0.4) | 5 (0.5) | 0 | 33 (2.7) | 31 (3.1) | 2 (0.9) | 86 (7.1) | 79 (7.9) | 7 (3.2) | 354 (29.0) | 288 (28.8) | 66 (30.0) | 741 (60.8) | 596 (59.7) | 145 (65.9) |
| Discussions are fruitful | 14 (1.1) | 14 (1.4) | 0 | 87 (7.1) | 84 (8.4) | 3 (1.4) | 156 (12.8) | 141 (14.1) | 15 (6.8) | 459 (37.7) | 380 (38.0) | 79 (35.9) | 503 (41.3) | 380 (38.0) | 123 (55.9) |
| Data are useful | 40 (3.3) | 35 (3.5) | 5 (2.3) | 110 (9.0) | 100 (10.0) | 10 (4.5) | 253 (20.8) | 215 (21.5) | 38 (17.3) | 510 (41.8) | 410 (41.0) | 100 (45.5) | 306 (25.1) | 239 (23.9) | 67 (30.5) |
| Meetings are well organized | 5 (0.4) | 5 (0.5) | 0 | 33 (2.7) | 32 (3.2) | 1 (0.5) | 100 (8.2) | 91 (9.1) | 9 (4.1) | 429 (35.2) | 352 (35.2) | 77 (35.0) | 652 (53.5) | 519 (52.0) | 133 (60.5) |
| Meeting duration is suitable | 15 (1.2) | 15 (1.5) | 0 | 49 (4.0) | 46 (4.6) | 3 (1.4) | 67 (5.5) | 63 (6.3) | 4 (1.8) | 414 (34.0) | 348 (34.8) | 66 (30.0) | 674 (55.3) | 527 (52.8) | 147 (66.8) |
| Practice data are discussed openly | 20 (1.6) | 19 (1.9) | 1 (0.5) | 56 (4.6) | 52 (5.2) | 4 (1.8) | 146 (12.0) | 125 (12.5) | 21 (9.5) | 409 (33.6) | 331 (33.1) | 78 (35.5) | 588 (48.2) | 472 (47.2) | 116 (52.7) |
| Experiences are shared | 8 (0.7) | 8 (0.8) | 0 | 18 (1.5) | 17 (1.7) | 1 (0.5) | 59 (4.8) | 53 (5.3) | 6 (2.7) | 383 (31.4) | 326 (32.6) | 57 (25.9) | 751 (61.6) | 595 (59.6) | 156 (70.9) |
| Agreement is easily reached | 25 (2.1) | 23 (2.3) | 2 (0.9) | 86 (7.1) | 73 (7.3) | 13 (5.9) | 344 (28.2) | 296 (29.6) | 48 (21.8) | 532 (43.6) | 419 (41.9) | 113 (51.4) | 232 (19.0) | 188 (18.8) | 44 (20.0) |
| Commitment is great | 24 (2.0) | 22 (2.2) | 2 (0.9) | 103 (8.4) | 83 (8.3) | 20 (9.1) | 292 (24.0) | 245 (24.5) | 47 (21.4) | 470 (38.6) | 384 (38.4) | 86 (39.1) | 330 (27.1) | 265 (26.5) | 65 (29.5) |

Table notes:

All GPs n=1219, ordinary cluster members (OCM) n=999 and Cluster leads (CL) including cluster coordinator and administrative ordinary cluster members n=220.

### Table A6. Self-reported benefits; answers divided by cluster membership status, 2020

|  | Very little n (%) | | | Little n (%) | | | Moderate n (%) | | | High n (%) | | | Very high n (%) | | |
| --- | --- | --- | --- | --- | --- | --- | --- | --- | --- | --- | --- | --- | --- | --- | --- |
|  | ALL | OCM | CL | ALL | OCM | CL | ALL | OCM | CL | ALL | OCM | CL | ALL | OCM | CL |
| Overall benefit | 123 (10.1) | 121 (12.1) | 2 (0.9) | 241 (19.8) | 210 (21.0) | 31 (14.1) | 488 (40.0) | 420 (42.0) | 68 (30.9) | 305 (25.0) | 209 (20.9) | 96 (43.6) | 62 (5.1) | 39 (3.9) | 23 (10.5) |
|  | None n (%) | | | Little n (%) | | | To some degree n (%) | | | To a high degree n (%) | | | Not relevant n (%) | | |
| Changes: |  |  |  |  |  |  |  |  |  |  |  |  |  |  |  |
| Clinical organization and workflow | 366 (30.0) | 335 (33.5) | 31 (14.1) | 398 (32.6) | 31 (14.1) | 68 (30.9) | 365 (29.9) | 267 (26.7) | 98 (44.5) | 61 (5.0) | 41 (4.1) | 20 (9.1) | 29 (2.4) | 26 (2.6) | 3 (1.4) |
| Drug prescriptions | 251 (20.6) | 225 (22.5) | 26 (11.8) | 401 (32.9) | 31 (14.1) | 60 (27.3) | 408 (33.5) | 315 (31.5) | 93 (42.3) | 139 (11.4) | 104 (10.4) | 35 (15.9) | 20 (1.6) | 14 (1.4) | 6 (2.7) |
| Improvements: |  |  |  |  |  |  |  |  |  |  |  |  |  |  |  |
| Increased knowledge of guidelines | 379 (31.1) | 329 (32.9) | 50 (22.7) | 394 (32.3) | 331 (33.1) | 63 (28.6) | 339 (27.8) | 260 (26.0) | 79 (35.9) | 63 (5.2) | 45 (4.5) | 18 (8.2) | 44 (3.6) | 34 (3.4) | 10 (4.5) |
| Overall patientcare in the clinic | 259 (21.2) | 238 (23.8) | 21 (9.5) | 559 (45.9) | 465 (46.5) | 94 (42.7) | 328 (26.9) | 248 (24.8) | 80 (36.4) | 51 (4.2) | 32 (3.2) | 19 (8.6) | 22 (1.8) | 16 (1.6) | 6 (2.7) |

Table notes:

All GPs n=1219, ordinary cluster members (OCM) n=999 and Cluster leads (CL) including cluster coordinator and administrative ordinary cluster members n=220.

### Table A7. Associations between gained knowledge and how the clusters are perceived, based on answers from ordinary cluster members and

**cluster leads, 2020**

| Overall benefit | | | |
| --- | --- | --- | --- |
|  | Ordinary cluster members | Cluster leads |  |
| Perceived meeting experiences | Overall benefit | Overall benefit |  |
| Topics are relevant | 1.6 (1.0-2.7) | 3.2 (0.4-25.7) |  |
| Meetings are productive | 4.3 (2.8-6.8) | 10.7 (3.2-35.1) |  |
| Atmosphere is friendly | 0.9 (0.6-1.4) | 1.2 (0.2-5.6) |  |
| Discussions are fruitful | 2.5 (1.6-3.8) | 0.8 (0.2-3.1) |  |
| Data are useful | 1.9 (1.4-2.5) | 2.0 (0.9-4.6) |  |
| Meetings are well organized | 1.0 (0.6-1.7) | 0.3 (0.1-1.2) |  |
| Meeting duration is suitable | 2.9 (1.9-4.5) | 14.7 (2.0-108.3) |  |
| Practice data are discussed openly | 0.9 (0.6-1.3) | 3.0 (0.9-9.6) |  |
| Experiences are shared | 1.0 (0.6-1.9) | 0.7 (0.1-3.2) |  |
| Agreement is easily reached | 1.2 (0.9-1.5) | 1.8 (0.9-3.5) |  |
| Commitment is great | 2.8 (2.0-4.1) | 3.2 (1.5-6.8) |  |

Table notes:

Ordered logistic multivariable regression model with cluster robust standard errors at the quality cluster level. Adjusted for GP age and

gender, practice type, area, cluster organizational characteristics (frame: frequency, duration, and rules for attendance; content: use of

plenum discussions, groupwork, cluster packages, and guidelines), upstart date, size and degree of participation in meetings.

Missing values were allocated to the majority group for categorical variables: female, The Capital Region and the mean for continuous

variables: mean GP age was 51 years.

Ordinary cluster members n=999 and Cluster leads including cluster coordinator and administrative ordinary cluster members n=220.

### Table A8. Associations between gained knowledge and how the clusters are perceived, based on answers from ordinary cluster members and

**cluster leads, 2020**

| Change | | | | |
| --- | --- | --- | --- | --- |
|  | Ordinary cluster members | | Cluster leads |  |
| Perceived meeting experiences | Organization and workflow | Drug prescriptions | Organization and workflow | Drug prescriptions |
| Topics are relevant | 12.2 (0.7-212.8) | 1.3 (0.8-2.1) | 12.2 (0.7-212.8) | 6.6 (0.4-99.8) |
| Meetings are productive | 3.0 (1.0-9.0) | 2.4 (1.6-3.7) | 3.0 (1.0-9.0) | 1.3 (0.4-4.1) |
| Atmosphere is friendly | 0.6 (0.1-2.8) | 1.0 (0.6-1.5) | 0.6 (0.1-2.8) | 0.2 (0.0-1.4) |
| Discussions are fruitful | 0.8 (0.3-2.5) | 1.3 (0.9-1.9) | 0.8 (0.3-2.5) | 1.2 (0.4-4.1) |
| Data are useful | 1.8 (0.9-3.7) | 2.0 (1.5-2.7) | 1.8 (0.9-3.7) | 1.4 (0.7-2.9) |
| Meetings are well organized | 0.7 (0.2-3.3) | 0.8 (0.5-1.2) | 0.7 (0.2-3.3) | 0.8 (0.2-3.1) |
| Meeting duration is suitable | 0.9 (0.2-4.2) | 1.9 (1.3-2.9) | 0.9 (0.2-4.2) | 0.2 (0.0-0.8) |
| Practice data are discussed openly | 1.8 (0.4-7.9) | 1.0 (0.8-1.4) | 1.8 (0.4-7.9) | 2.5 (1.0-6.3) |
| Experiences are shared | 2.5 (0.5-13.3) | 1.3 (0.7-2.2) | 2.5 (0.5-13.3) | 1.5 (0.3-6.6) |
| Agreement is easily reached | 0.9 (0.4-1.8) | 1.1 (0.8-1.5) | 0.9 (0.4-1.8) | 2.4 (1.2-4.8) |
| Commitment is great | 2.0 (1.0-3.8) | 1.9 (1.3-2.6) | 2.0 (1.0-3.8) | 3.2 (1.6-6.4) |

Figure notes:

Ordered logistic multivariable regression model with cluster robust standard errors at the quality cluster level. Adjusted for GP age and

gender, practice type, area, cluster organizational characteristics (frame: frequency, duration, and rules for attendance; content: use of

plenum discussions, groupwork, cluster packages, and guidelines), upstart date, size and degree of participation in meetings.

Missing values were allocated to the majority group for categorical variables: female, The Capital Region and the mean for continuous

variables: mean GP age was 51 years.

Ordinary cluster members n=999 and Cluster leads including cluster coordinator and administrative ordinary cluster members n=220.

### Table A9. Associations between gained knowledge and how the clusters are perceived, based on answers from ordinary cluster members and

**cluster leads, 2020**

| Improvements | | | | |
| --- | --- | --- | --- | --- |
|  | Ordinary cluster members | | Cluster leads | |
| Perceived meeting experiences | Knowledge of guidelines | Knowledge of guidelines | Knowledge of guidelines | Overall patient care in the clinic |
| Topics are relevant | 1.5 (1.0-2.3) | 1.5 (1.0-2.3) | 5.1 (0.3-94.9) | 1.6 (0.4-5.7) |
| Meetings are productive | 1.8 (1.3-2.6) | 1.8 (1.3-2.6) | 3.3 (0.7-14.7) | 1.5 (0.5-4.9) |
| Atmosphere is friendly | 0.8 (0.5-1.2) | 0.8 (0.5-1.2) | 0.4 (0.1-2.9) | 3.1 (0.3-35.8) |
| Discussions are fruitful | 1.7 (1.2-2.5) | 1.7 (1.2-2.5) | 0.6 (0.1-2.8) | 4.2 (1.1-16.3) |
| Data are useful | 1.3 (1.0-1.8) | 1.3 (1.0-1.8) | 1.6 (0.7-3.5) | 2.2 (0.8-5.9) |
| Meetings are well organized | 0.8 (0.5-1.1) | 0.8 (0.5-1.1) | 0.3 (0.1-1.0) | 0.5 (0.1-2.1) |
| Meeting duration is suitable | 1.3 (0.8-2.0) | 1.3 (0.8-2.0) | 3.1 (0.3-35.8) | 0.8 (0.3-2.3) |
| Practice data are discussed openly | 1.2 (0.9-1.6) | 1.2 (0.9-1.6) | 0.9 (0.3-2.8) | 3.3 (0.9-11.3) |
| Experiences are shared | 1.0 (0.6-1.7) | 1.0 (0.6-1.7) | 14.6 (2.2-96.1) | 4.3 (1.0-19.4) |
| Agreement is easily reached | 1.2 (0.9-1.5) | 1.2 (0.9-1.5) | 1.2 (0.6-2.5) | 1.7 (0.8-3.7) |
| Commitment is great | 1.8 (1.3-2.5) | 1.8 (1.3-2.5) | 1.1 (0.6-2.1) | 2.5 (1.2-4.8) |

Figure notes:

Ordered logistic multivariable regression model with cluster robust standard errors at the quality cluster level. Adjusted for GP age and

gender, practice type, area, cluster organizational characteristics (frame: frequency, duration, and rules for attendance; content: use of

plenum discussions, groupwork, cluster packages, and guidelines), upstart date, size and degree of participation in meetings.

Missing values were allocated to the majority group for categorical variables: female, The Capital Region and the mean for continuous

variables: mean GP age was 51 years.

Ordinary cluster members n=999 and Cluster leads including cluster coordinator and administrative ordinary cluster members n=220.
